# Synchrotron-based infrared microspectroscopy unveils the biomolecular response of healthy and tumour cell lines to neon minibeam radiation therapy†

**DOI:** 10.1039/d4an01038h

**Published:** 2024-12-13

**Authors:** R. González-Vegas, O. Seksek, A. Bertho, J. Bergs, R. Hirayama, T. Inaniwa, N. Matsufuji, T. Shimokawa, Y. Prezado, I. Yousef, I. Martínez-Rovira

**Affiliations:** a Physics Department, Universitat Autònoma de Barcelona (UAB) 08193 Cerdanyola del Vallès Barcelona Spain Immaculada.Martinez@uab.cat; b IJCLab, French National Centre for Scientific Research 91450 Orsay France; c Institut Curie, Université PSL, CNRS UMR3347, Inserm U1021, Signalisation Radiobiologie et Cancer 91400 Orsay France; d Université Paris-Saclay, CNRS UMR3347, Inserm U1021, Signalisation Radiobiologie et Cancer 91400 Orsay France; e Radiology Department, Charité-Universitätsmedizin Berlin 10117 Berlin Germany; f Department of Charged Particle Therapy Research, Institute for Quantum Medical Science, National Institutes for Quantum Science and Technology (QST) 4-9-1 Anagawa Inage-ku Chiba-shi 263-8555 Japan; g Department of Accelerator and Medical Physics, QST 4-9-1 Anagawa Inage-ku Chiba-shi 263-8555 Japan; h New Approaches in Radiotherapy Lab, Center for Research in Molecular Medicine and Chronic Diseases (CIMUS), Instituto de Investigación Sanitaria de Santiago de Compostela (IDIS), University of Santiago de Compostela Santiago de Compostela A Coruña 15706 Spain; i Oportunius Program, Galician Agency of Innovation (GAIN), Xunta de Galicia, Santiago de Compostela A Coruña Spain; j MIRAS Beamline, ALBA Synchrotron 08209 Cerdanyola del Vallès Barcelona Spain

## Abstract

Radioresistant tumours remain complex to manage with current radiotherapy (RT) techniques. Heavy ion beams were proposed for their treatment given their advantageous radiobiological properties. However, previous studies with patients resulted in serious adverse effects in the surrounding healthy tissues. Heavy ion RT could therefore benefit from the tissue-sparing effects of minibeam radiation therapy (MBRT). To investigate the potential of this combination, here we assessed the biochemical response to neon MBRT (NeMBRT) through synchrotron-based Fourier transform infrared microspectroscopy (SR-FTIRM). Healthy (BJ) and tumour (B16-F10) cell lines were subjected to seamless (broad beam) neon RT (NeBB) and NeMBRT at HIMAC. SR-FTIRM measurements were conducted at the MIRAS beamline of ALBA Synchrotron. Principal component analysis (PCA) permitted to assess the biochemical effects after the irradiations and 24 hours post-irradiation for the different RT modalities and doses. For the healthy cells, NeMBRT resulted in the most dissimilar spectral signatures from non-irradiated cells early after irradiations, mainly due to protein conformational modifications. Nevertheless, most of the damage appeared to recover one day post-RT; conversely, protein- and nucleic acid-related IR bands were strongly affected by NeBB 24 hours after treatment, suggesting superior oxidative damage and nucleic acid degradation. Tumour cells appeared to be less sensitive to NeBB than to NeMBRT shortly after RT. Still, after one day, both NeBB and the high-dose NeMBRT regions yielded important spectral modifications, suggestive of cell death processes, protein oxidation or oxidative stress. Lipid-associated spectral changes, especially due to the NeBB and NeMBRT peak groups for the tumour cell line, were consistent with reactive oxygen species attacks.

## Abstract 2

The biochemical impact of neon minibeam radiation therapy (NeMBRT) on BJ (healthy) and B16-F10 (tumour) cell lines was investigated through synchrotron-based infrared microspectroscopy.

## Graphical abstract

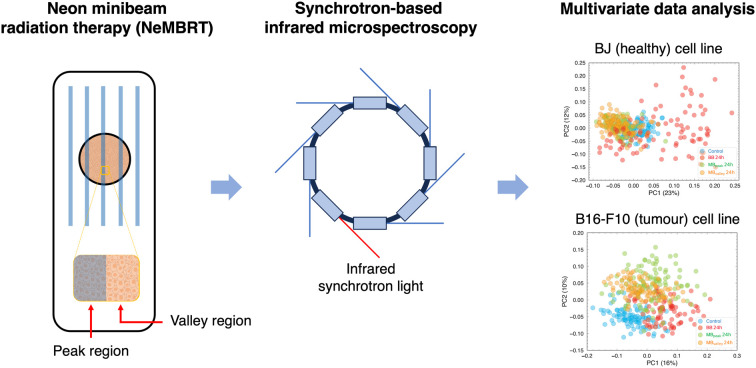

## 1 Introduction

Cancer is one of the leading causes of death in the world. Accordingly, radiotherapy (RT) has established itself as one of the most important therapeutic options against these diseases: approximately 50–70% of cancer patients will receive RT treatment during the duration of their disease. The last decades have seen significant technological advances in RT, related to greater precision in tumour delineation, improved dose conformation and reduced toxicities to normal tissues, all of which have led to better treatment outcomes. Still, the management of some cancer variants remains difficult to address with conventional RT techniques. An example would be the treatment of radioresistant tumours, which are generally associated with a poor prognosis.

For that matter, the use of heavy ion beams (*e.g.* carbon or neon beams) was proposed to cope with these challenging scenarios. They exhibit a superior biological effectiveness and a lower oxygen enhancement ratio with respect to other beams, suggesting heavy ions to be adequate for the treatment of radioresistant malignancies. In the period between 1977 and 1992, a few hundred cancer patients (including disease types such as malignant gliomas, salivary gland carcinomas, bone sarcomas, and others) received neon RT, in some cases combined with photon or helium RT.^1,2^ However, some patients experienced severe adverse effects, which led to the cessation of the use of neon beams in a clinical setting.

The quest for novel RT modalities that widen the therapeutic window has resulted in a paradigm shift from the traditional approaches, leading to changes in temporal schemes, dose rates or spatial dose distributions. Concerning the latter point, minibeam radiation therapy (MBRT) has emerged as a promising alternative to conventional RT. Firstly proposed in 2006,^3^ MBRT consists in a spatial dose modulation, employing arrays of beamlets, 0.5–1.0 mm wide and separated by a center-to-center (c-t-c) distance of 1–4 mm. These beam characteristics have been shown to increase normal tissue dose tolerances using several types of beams.^4–6^ Regarding tumour control, MBRT has proven to be, at least, as equally effective as conventional RT.^6–8^

In this context, a promising reinvented use of neon beams could be realised in combination with MBRT (NeMBRT). Previous Monte Carlo studies evaluated the dosimetric feasibility of this technique.^9,10^ Their findings showed that NeMBRT could provide a high peak-to-valley dose ratio (PVDR) with low valley doses and reduced linear energy transfer (LET) values in normal tissues (favourable for their sparing). These results guided a subsequent biological study in which the normal tissue response to seamless (broad beam) neon RT (NeBB) and NeMBRT was evaluated.^11^ Severe damage was observed in mice subjected to NeBB, including cutaneous ulceration and epidermal necrosis. On the contrary, NeMBRT-treated mice only presented mild hair loss and erythema without ulceration. Thus, authors concluded that NeMBRT offered a gain in healthy tissue preservation compared to NeBB, regardless of the high peak doses.

Despite these promising results, the full biological basis of NeMBRT (and MBRT in general) has yet to be fully disentangled. The main mechanisms suggested to explain the response of both healthy and tumour tissues to these novel RT approaches are: differential vascular effects, particularly the great impact of spatial dose fractionation on immature vessels;^12^ the migration of stem cells from valley to peak dose regions in normal tissues in favour of repair processes;^13^ the immune system activation as antitumour response;^14^ cell signalling mechanisms (*e.g.* bystander/cohort and abscopal effects);^15,16^ and the direct and indirect effects of reactive oxygen species (ROS).^17^

One possible approach to grasp the remaining biochemical mechanisms underlying MBRT would be having recourse to synchrotron radiation-based Fourier transform infrared microspectroscopy (SR-FTIRM). This non-destructive modality is based on the vibrational excitation of molecular bonds due to their interaction with infrared (IR) light. SR-FTIRM is an excellent tool for interrogating biological materials and studying their biochemical structure and modifications without altering them.^18^ Also, the use of synchrotron IR light provides an excellent signal-to-noise ratio with the high spatial resolution required in single-cell analyses. SR-FTIRM has proven useful to uncover the biomolecular response to innovative RT modalities, such as proton therapy,^19,20^ RT combined with nanoparticles,^21–23^ X-ray microbeam RT,^24^ proton MBRT (pMBRT)^25^ or FLASH-RT.^26^

Therefore, and given that the alliance of heavy ions with MBRT is a promising alternative to current therapeutic practices for certain treatments, the present SR-FTIRM study is aimed at providing new insights into the biomolecular rationale that underlies NeMBRT. To this end, both healthy and tumour cell lines subjected to NeBB and NeMBRT were evaluated through SR-FTIRM at different doses and post-irradiation time-points.

## 2 Experimental section

### 2.1 Sample preparation & irradiations

BJ human foreskin fibroblasts (ATCC®-CRL-2522™) and B16-F10 mouse skin melanoma (ATCC®-CRL-6475™) cell lines were purchased from ATCC. Both cell lines were cultured in high glucose DMEM medium (Gibco™, LifeTechnologies SAS, Courtaboeuf, France) supplemented with 10% fetal calf serum, 1% penicillin–streptomycin (10 000 units per mL each), 1 mM GlutaMAX™, 1 mM sodium pyruvate and 10 mM HEPES. The conditions of the incubation chamber were set to 37 °C, 95% humidity and 5% CO_2_. Samples were directly grown onto 0.5 mm-thick IR transparent CaF_2_ coverglasses (Crystran Ltd) attached to the slides of Thermo Scientific™Nunc™Lab-Tek™ flasks; 1 mL of cell suspension was seeded in each coverglass (at a concentration of 5 × 10^4^ cells per mL) so as to obtain a 75–80% confluence rate on the day of irradiations.

**Fig. 1:**
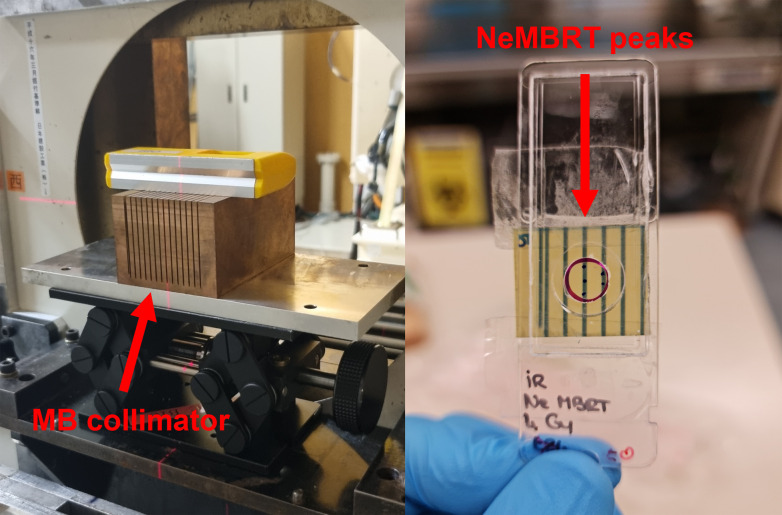
Photograph of the beamline in the biology room of HIMAC, where RT irradiations were performed; the multislit brass collimator for minibeam generation can be seen (left). Radiochromic films were attached to the back of the IR slides containing the cells; the peak and valley regions are clearly visible after NeMBRT irradiations (right).

RT experiments were carried out at the Heavy-Ion Medical Accelerator in Chiba (HIMAC, National Institutes for Quantum Science and Technology, Chiba, Japan). At HIMAC, heavy ion species ranging from helium to argon can be accelerated for their medical use.^27^ Neon beams of 230 MeV/u (energy at the exit of the accelerator) and LET of 45 keV/μm (value at the target position^28^) were used to perform NeBB and NeMBRT irradiations, delivering mean (physical) doses of 2, 4 and 8 Gy. In spatially fractionated RT, the previous values refer to the mean doses of NeMBRT lateral profiles. The specific peak and valley doses were (respectively): 6.9 ± 0.7 Gy and 0.11 ± 0.01 Gy (2 Gy mean dose), 16 ± 2 Gy and 0.20 ± 0.02 Gy (4 Gy mean dose), and 29 ± 3 Gy and 0.35 ± 0.04 Gy (8 Gy mean dose). Minibeams were generated by means of a divergent 10 cm-thick multislit brass collimator, attached at the exit of the accelerator (Fig. 1). Slits were 700 μm-wide and they were separated by a c-t-c distance of 3500 μm. Dosimetry was accomplished by using Gafchromic™EBT3 films attached to the IR slides containing the samples, which allowed to guarantee irradiation quality, to localize the NeBB field, and to distinguish between NeMBRT peak and valley regions (also allowing to select the cells corresponding to these groups during SR-FTIRM measurements, see Fig. 1).

The cell lines were fixated at two different time-points post-RT: following irradiations (henceforth labelled as 0h) and 24 hours later (henceforth labelled as 24h). Early after RT, medium of one half of the flasks containing the samples was removed and slides were rinsed twice with phosphate-buffered saline. Subsequently, samples were incubated for 1 hour at room temperature with 10% formalin neutral buffered solution (Sigma-Aldrich). Then, any residual phosphate ions were washed out after 3 rounds of ultrapure water rinsing, and samples were dried out at room temperature for posterior SR-FTIRM analyses. The other half of the flasks were incubated for one day, after which the same procedure as described above was applied for their fixation.

### 2.2 SR-FTIRM measurements

Samples subjected to both RT treatments were submitted to SR-FTIRM measurements at the MIRAS beamline of ALBA Synchrotron. The end-station is equipped with a Hyperion 3000 microscope coupled to a Vertex 70 spectrometer (Bruker Optics GmbH, Germany). A mercury cadmium telluride detector, cooled with liquid nitrogen, enabled the acquisition of the IR data. Single masking aperture sizes (IR beam size) were set to 16 × 16 μm^2^ (BJ cell line) and 9 × 9 μm^2^ (B16-F10 cell line) for single cell measurements; the different aperture sizes were chosen due to the size differences between the two cell lines. Over 125 cells were randomly selected from each sample and irradiation condition (Control, BB, MB_peak_ and MB_valley_). IR spectra of the cells were acquired using the transmission measurement mode of the microscope. Single cell IR measurements were collected in the 3800–1000 cm^−1^ mid-IR range, with a spectral resolution of 4 cm^−1^; 256 scans coupled with 40 kHz scanning velocity lead to an exposure time of 1 min per spectrum. Background spectra were collected every 5 samples to compensate for varying ambient conditions in the beamline during the measurements, under the same acquisition parameters as previously described.

### 2.3 Data analysis

**Fig. 2:**
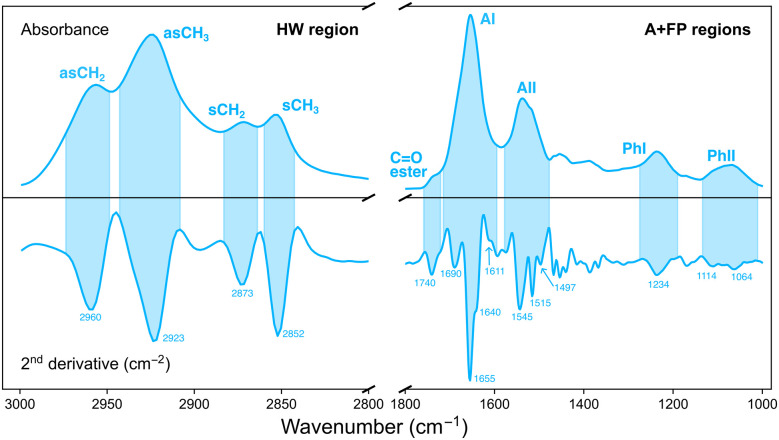
IR absorbance spectrum (top) of a cell and its second derivative (bottom) in the HW (left) and A + FP (right) spectral regions. Coloured areas indicate the spectral range of the indicated IR bands for both spectra. The position (in cm^−1^) of the minima of the most relevant IR bands are indicated. The absorbance spectrum was baseline corrected and vector normalised; the second derivative spectrum was vector normalised.

Analysis of the IR data was performed with the open source software Quasar (version 1.9).^29^ Principal component analysis (PCA) was used as an unsupervised, multivariate method to investigate the effect of the various irradiation configurations on samples according to their different IR biochemical signatures. PCA was performed in two separate spectral regions (Fig. 2):

• Amides and fingerprint (A + FP, 1800–1000 cm^−1^). The 1800–1400 cm^−1^ range originates from vibrational modes of proteins and peptides,^30^ and is composed of two main IR bands: the 1710–1590 cm^−1^ spectral range is associated to the Amide I (AI) band and arises from C=O stretching vibrations and, to a lesser extent, from out-of-phase CN stretching, CCN deformation and NH in-plane bending; the 1585–1478 cm^−1^ spectral region is assigned to the Amide II (AII) band, arising from in-plane NH bending and CN stretching vibrations. The low-frequency region (1350–1000 cm^−1^) results from carbohydrates and sugar-phosphate vibrations, providing information on the conformations of the nucleic acids backbone.^31^

• Higher wavenumber (HW, 3000–2800 cm^−1^). Arises from stretching vibrations of C–H groups (methyl and methylene), present in the hydrocarbon acyl chains of membrane lipids.^32^

Multivariate analysis in each region was performed on second-derivative, vector normalised IR spectra. Differentiation was accomplished by using a Savitzky–Golay filter (9 points window for the HW region; 19–25 points window for the A + FP regions) and made it possible to overcome baseline artifacts in the data, as well as to resolve overlapping IR bands.^25,33^ Prior to PCA, second-derivative IR spectra were unit vector normalised.

Additionally, violin plots showing the probability density of several spectral band ratios of interest were generated for both cell lines, used as markers of biochemical modifications: the Phosphate I (1280–1185 cm^−1^) to Amide II (1585–1478 cm^−1^), PhI/AII; the Phosphate II (1140–1010 cm^−1^) to Amide II (1585–1478 cm^−1^), PhII/AII; the asymmetric methylene (2948–2900 cm^−1^) to asymmetric methyl (2978–2947 cm^−1^), asCH_2_/asCH_3_; and the carbonyl ester (1760–1725 cm^−1^) to asymmetric methyl (2978–2947 cm^−1^), C=O/asCH_3_. The Kruskal–Wallis test was employed to assess the global significance between groups and, if differences were statistically significant, a Dunn test with the Bonferroni adjustment was used to perform pairwise comparisons. Statistical analysis was conducted with the software R (version 4.3.2).^34^

## 3 Results & discussion

PCA results for samples fixated at 0h and 24h post-irradiations are presented in sections 3.1 and 3.2, respectively. Results for the A + FP and HW spectral regions are reported separately. Discussion covers both the BJ and B16-F10 cell lines irradiated with the three doses studied. Data for the intermediate dose of 4 Gy (Fig. S1†), as well as the figures with the average absorbance spectra for all irradiation configurations (Fig. S2 and S3†) are included in the ESI.†

### 3.1 PCA at 0h post-RT

#### A + FP regions

**Fig. 3:**
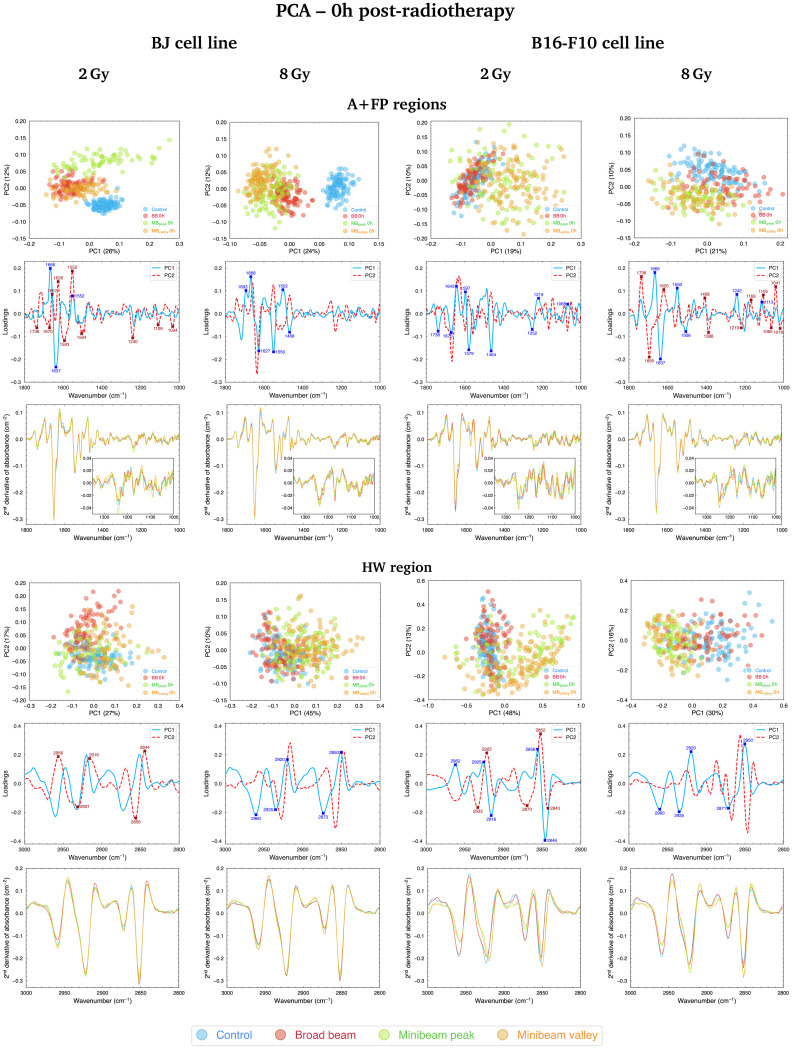
PCA in the A + FP (1800–1000 cm^−1^, top) and HW (3000–2800 cm^−1^, bottom) spectral regions of BJ (left) and B16-F10 (right) cells fixated at 0h post-RT (2 and 8 Gy); for each spectral region, the PCA scores (upper row), loadings (middle row) and average second-derivative absorbance spectra (lower row) are included. Each point of the PCA scores is a cell spectrum, and colours correspond to the irradiation configurations: blue for Control (non-irradiated), red for BB, green for MB_peak_ and orange for MB_valley_. Explained variances by the PCs are included in parentheses. In the loadings, the contribution of each spectral band to data separation along PC1 is indicated by solid blue lines, while the bands contributing to the separation along PC2 are indicated by dashed red lines. The most relevant IR peaks contributing to the cluster delineation along PC1 or PC2 are indicated with blue or red labels and crosses, respectively. Indicated doses refer to the mean dose for both NeBB and NeMBRT configurations.

Fig. 3 (top, left; 2 Gy and 8 Gy) and Fig. S1† (top, left; 4 Gy) show the PCA results for BJ cells fixated at 0h post-treatment. PCA score plots delineate the clustering of samples according to the spectral differences between the irradiation modalities, and the loadings help identifying the main IR bands that contribute to data separation. PCA scores show irradiated groups well separated from Control cells. Differences between NeBB and NeMBRT groups can also be noticed, as well as between the peak and valley regions of NeMBRT. The peaks with the highest scores in the loadings (contributing the most to cluster separation) for the three doses are encountered in the spectral ranges associated with the AI and AII bands. The amide bands can provide information about the different protein secondary structures, and their alterations might be indicative of protein conformational disorders.^30^ Specific substructures seem to have suffered alterations after RT: the α-helix (1680–1634 cm^−1^) and β-sheet (1658–1618 cm^−1^) of AI^35^ (although the latter range might also account for contributions from a random coil of AI), and the α-helix (1565–1520 cm^−1^) and β-sheet of AII (1540–1495 cm^−1^);^36^ the contribution near 1668 cm^−1^ arise mainly from the AI α-helix coupled with a minor contribution from the AI β-turn (1695–1660 cm^−1^).^37^ Modifications of these bands due to the various irradiation modalities are dose-dependent, but the loadings reveal that both the AI α-helix and β-sheet bands have a great importance in differentiating Control from irradiated groups.

Regarding the changes in the FP region, the main contributions to data separation are encountered in the 1250–1220 cm^−1^ spectral range, arising from asymmetric stretching vibrations of the PO_2_^−^ band, named PhI (1280–1185 cm^−1^).^38^ Changes in this band were dose-dependent and primarily contributed to differentiate Control and RT-treated samples, but also helped to separate NeBB and NeMBRT groups. PhI modifications are considered indicative of RT-induced DNA damage^39^ and could be related to strand cleavage and chromatin fragmentation due to DNA breakages,^19^ DNA condensation and degradation,^40^ or oxidative stress.^41^ Additional bands in the low-frequency region also contribute to data splitting, with the main ones being the A-form DNA (1180–1160 cm^−1^)^38^ and the 1140–1010 cm^−1^ spectral range, named PhII.^38^ The latter region mainly arises from PO_2_^−^ symmetric stretching modes and C–O furanose vibrations. Modifications of these bands might be indicative of DNA-associated alterations upon RT modalities, such as rearrangements of nucleic acids structures,^42^ increased DNA breakages,^43^ and base stacking and pairing alterations.^44^ These changes are mainly associated to the BB and MB_peak_ groups.

PCA results in the A + FP regions for the B16-F10 cell line fixated at 0h post-treatment are shown in Fig. 3 (top, right; 2 Gy and 8 Gy) and Fig. S1† (top, left; 4 Gy). NeBB remains proximal to Control, while NeMBRT clusters depart from these groups. For the 2 Gy, the main peaks explaining the separation of NeMBRT clusters are assigned to the C=O carbonyl ester band (1760–1725 cm^−1^, arising from stretching vibrations of cellular phospholipids^41^), substructures of AI and AII, including the 1600–1589 cm^−1^ spectral region arising from NH_2_ vibrations of amine groups,^37^ and the CH_2_ bending modes of the acyl chains of lipids (peak near 1464 cm^−1^),^30^ as well as to several contributions in the FP region from the PhI and PhII bands. The peak near 1579 cm^−1^ is assigned to in-plane bending vibrations of the NH_2_ group of AII and the ring of the adenine and cytosine, and could reflect a degree of damage to these base pairs.^39^ Most of the intra-group variability of the MB_peak_ and MB_valley_ groups comes from differences in the β-turn, α-helix and β-sheet substructures of AI. Regarding the intermediate and high doses, most of the observed separation of RT-treated groups from Control is due to contributions from the anti-parallel β-sheet (1705–1685 cm^−1^)^37^ and the β-turn of AI, the α-helix of AII, the PhI band (mainly from contributions of the B-form DNA in the 1225–1220 cm^−1^ range^38^), the symmetric phosphodiester stretching of the DNA backbone and the C–O furanose. These spectral features, particularly those in the FP region, mainly contribute to the separation of NeMBRT peak and valley groups from the other clusters. Additionally, the peaks in the 1410–1385 cm^−1^ spectral region arise from complex vibrational modes of the COO− and CH_3_ groups present in the fatty acids, proteins and amino acids;^36,45^ alterations in this region are associated with the effects of the MB_peak_ and MB_valley_ groups.

Comparison of cluster separation for the two cell lines revealed some key differences in the response to treatment modalities at this time-point. The healthy cell line resulted sensitive to both types of treatment: both NeBB and NeMBRT clusters showed clearly distinct from Control group. Also, some differences between MB_peak_ and MB_valley_ were noticeable at this time-point. On the contrary, the tumour cell line seemed less sensitive to NeBB, with this group being quite close to Control for the three doses, whereas NeMBRT groups were already well distinct from the Control cluster as for the healthy cell line. The relative contribution of the FP spectral region (with respect to the amides region) to data segregation also seemed to be greater for the tumour cell line than for the healthy one.

#### HW region

Fig. 3 (bottom, left; 2 Gy and 8 Gy) and Fig. S1† (bottom, left; 4 Gy) show the PCA in the HW region for BJ cells fixated at 0h post-RT. Different separation trends can be observed as a function of the dose. NeBB separates from Control and NeMBRT groups for 2 Gy; for 4 Gy, all RT modalities are slightly spreading from the Control group; and for 8 Gy, spectral differences between groups resulted in NeMBRT clusters separating from Control and NeBB samples. Differences between groups are less marked with respect to the A + FP region. The four main C–H stretching modes present in this spectral region were altered by RT modalities: the asymmetric (2978–2947 cm^−1^) and symmetric (2880–2863 cm^−1^) methyl stretching vibrations (asCH_3_ and sCH_3_, respectively), and the asymmetric (2948–2900 cm^−1^) and symmetric (2860–2840 cm^−1^) methylene stretching vibrations (asCH_2_ and sCH_2_, respectively). Most of the intra-group variability seems to be due to modifications of the asCH_2_ and sCH_2_ bands. The biggest cluster separation is encountered for the 8 Gy, mainly resulting from modifications of the CH_3_ stretching modes due to both MB_peak_ and MB_valley_ groups.

PCA results for the B16-F10 cell line in the HW region early after irradiations are depicted in Fig. 3 (bottom, right; 2 Gy and 8 Gy) and Fig. S1† (bottom, left; 4 Gy). The treatment effects are in the same line for the three doses: Control and NeBB groups are proximate to each other, while NeMBRT clusters spread away from them. Inspection of the loadings indicates that the main bands modified due to NeMBRT are the asCH_3_, sCH_3_ and sCH_2_. Specifically, the effects of NeMBRT on the methyl and methylene modes resulted in an intensity increase of the asCH_2_/asCH_3_ spectral band ratio (data not shown), and was already observed in cells with longer acyl chain lengths as a consequence of oxidative stress processes.^32,46^

### 3.2 PCA at 24h post-RT

#### A + FP regions

**Fig. 4:**
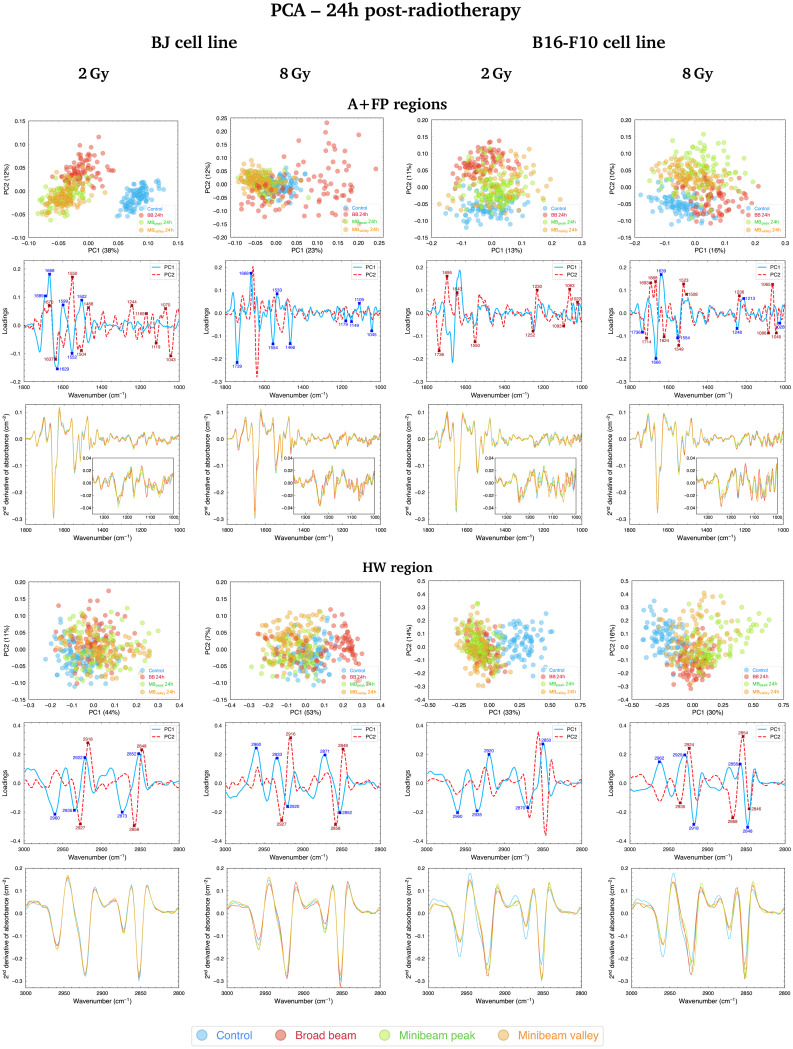
PCA in the A + FP (1800–1000 cm^−1^, top) and HW (3000–2800 cm^−1^, bottom) spectral regions of BJ (left) and B16-F10 (right) cells fixated at 24h post-RT (2 and 8 Gy); for each spectral region, the PCA scores (upper row), loadings (middle row) and average second-derivative absorbance spectra (lower row) are included. Each point of the PCA scores is a cell spectrum, and colours correspond to the irradiation configurations: blue for Control (non-irradiated), red for BB, green for MB_peak_ and orange for MB_valley_. Explained variances by the PCs are included in parentheses. In the loadings, the contribution of each spectral band to data separation along PC1 is indicated by solid blue lines, while the bands contributing to the separation along PC2 are indicated by dashed red lines. The most relevant IR peaks contributing to the cluster delineation along PC1 or PC2 are indicated with blue or red labels and crosses, respectively. Indicated doses refer to the mean dose for both NeBB and NeMBRT configurations.

**Fig. 5:**
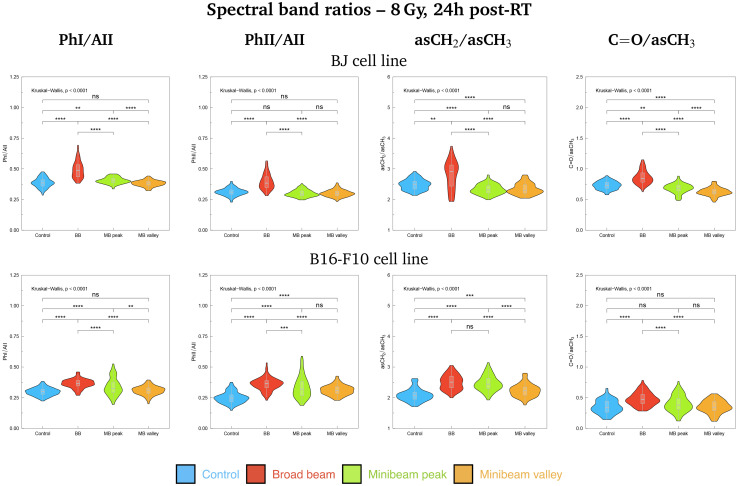
Violin plots showing the probability density distribution of the PhI/AII, PhII/AII, asCH_2_/asCH_3_ and C=O/asCH_3_ spectral band ratios for BJ (top) and B16-F10 (bottom) cell lines irradiated with 8 Gy and fixated at 24h post-RT. Colours correspond to the irradiation configurations: blue for Control (non-irradiated), red for BB, green for MB_peak_ and orange for MB_valley_. *p*-value significance levels are indicated as: (*p* > 0.05), *(*p* ≤ 0.05), **(*p* ≤ 0.01), ***(*p* ≤ 0.001), ****(*p* ≤ 0.0001). Indicated doses refer to the mean dose for both NeBB and NeMBRT configurations.

A different trend compared to results early after irradiations is observed for BJ cells fixated at 24h post-RT, depicted in Fig. 4 (top, left; 2 Gy and 8 Gy) and Fig. S1† (top, right; 4 Gy): NeMBRT groups stay closer to the Control cluster than NeBB, which is separating the most from non-irradiated cells; for the lowest dose, NeMBRT and NeBB groups remain well separated from the Control group, but NeBB is still the most dissimilar cluster compared to the non-irradiated sample. The contribution of the C=O carbonyl ester spectral range is particularly relevant in the separation of NeBB for the highest dose. Alterations of this band have been previously identified as hallmarks of cell death and oxidative damage,^47^ supported by the intensity increase of the C=O/asCH_3_ spectral band ratio^41^ for NeBB (Fig. 5, top). On the other hand, the MB_peak_ and MB_valley_ groups generally resulted in a decrease of the C=O/asCH_3_ ratio with respect to Control. Other bands, associated to the proteins, are also taking an important role in data splitting: the β-turn, α-helix, and β-sheet substructures of AI. These bands mainly contribute to segregate NeBB from the rest of the groups for the intermediate and high doses. Changes in cellular proteins are often associated with modifications in their distribution during cell death, oxidative stress, or to denaturation/oxidation of existing proteins,^24,47^ also in agreement with the observed alterations of the C=O carbonyl ester group due to NeBB. Additional bands were also particularly affected by NeBB, such as the α-helix of AII or the CH_2_ bending modes; modifications of the latter band have been associated with an altered conformation of lipid chain packing.^24^

Considering the modifications in the FP region, important contributions to data separation are encountered. For 2 Gy, conformational changes in the 1250–1040 cm^−1^ spectral range resulted in a separation between NeBB and NeMBRT clusters, with contributions from bands associated to the A-form DNA (peaks near 1240 cm^−1^ and 1170 cm^−1^), the ribose stretching (peak near 1115 cm^−1^) and the C–O stretching modes of the nucleic acids backbone and furanose (1070–1035 cm^−1^).^38,39^ The mentioned bands primarily contribute to the separation of the NeBB group; NeMBRT peak and valley IR signatures in this low-frequency region were closer to those of the Control. These band modifications reflect a degree of conformational changes and rearrangements in the structure of the nucleic acids after NeBB treatment, and might have resulted from DNA degradation or condensation,^40^ base alterations in the RNA,^44^ or even oxidative damage.^41^ Loadings for the 4 Gy and 8 Gy also indicate a contribution from the 1250–1000 cm^−1^ spectral region, mainly contributing to NeBB segregation from the other clusters. Interestingly, NeBB spectra exhibit a high intensity increase of the PhI/AII and PhII/AII spectral band ratios for the 8 Gy irradiations (Fig. 5, top), previously associated to increased DNA single- and double-strand breaks,^19^ or to oxidative stress.^41^

Cluster separation after PCA followed clear trends for BJ cells fixated at the two analysed time-points. Early after irradiations, NeMBRT-treated cells showed the most dissimilar IR signatures compared to the Control group. Conversely, the analysis at 24h post-RT revealed that the irradiation-induced damage due to NeBB appeared to be more persistent than that due to NeMBRT; this was reflected in the MB_peak_ and MB_valley_ groups being closer to the non-irradiated cells. Also, differences between MB_peak_ and MB_valley_ dose regions one day post-treatment were much more less noticeable than early after RT. At 24h, certain spectral markers became more important than at 0h for the segregation of NeBB from the other groups: the 1045–1035 cm^−1^ spectral region (C–O stretching modes of nucleic acids backbone and furanose); the band near 1637 cm^−1^ assigned to the AI β-sheet; and the C=O carbonyl ester band near 1740 cm^−1^, particularly for the highest dose. These spectral signatures might reflect unrecovered oxidative damage due to NeBB one day after irradiations;^41,47^ conversely, NeMBRT clusters being close to Control at 24h post-treatment reflect that a certain degree of the radiation-induced damage has already been recovered.

Fig. 4 (top, right; 2 Gy and 8 Gy) and Fig. S1† (top, right; 4 Gy) show the PCA for B16-F10 cells in the A + FP regions, where the three RT groups separate from the non-irradiated sample; it is noteworthy that for 8 Gy irradiations there is minimal overlap between groups, with MB_peak_ being less proximate to Control than the rest of configurations. The main spectral bands contributing to group separation are assigned to the C=O carbonyl ester, substructures of the AI and AII, the PhI and PhII bands, and the C–O furanose vibrations of Z-form DNA (1030–1015 cm^−1^).^38^ Modifications of the C=O carbonyl ester band contribute to the separation of the irradiated groups from Control, especially of the NeBB group, suggesting superior oxidative damage;^47^ again, an increase of the C=O/asCH_3_ spectral band ratio (Fig. 5, bottom) is consistent with cells being under oxidative stress. Additionally for 8 Gy irradiations, the appearance of peak near 1714 cm^−1^ in the spectral region associated to the C=O ester suggests that this group is becoming non-hydrogen bonded after oxidative damage,^36,47^ and contributes to differentiate NeMBRT from Control and NeBB clusters. Protein modifications essentially explain the separation of NeBB for the lowest dose and of MB_peak_ for the highest dose from the rest of the groups, with the anti-parallel β-sheet, β-turn and α-helix of AI, as well as the α-helix of AII, highly contributing to the separation of these groups. This might be suggestive of different conformational modifications after irradiations, such as protein oxidation.^36^ Spectral variations of the sugar-phosphate backbone bands in the low-frequency region by the BB and MB_peak_ groups are also consistent with oxidative stress.^36,45^ Regarding the PhI/AII and PhII/AII spectral band ratios (Fig. 5, bottom), an intensity increase for all irradiated groups compared to Control was detected. This behaviour is consistent with the results observed for pMBRT irradiations in a different tumour cell line,^25^ and might result from strand or chromatin cleavage after DNA fragmentation, or from oxidative stress.^19,41^

Comparing the two time-points analysed, we observed that NeBB-treated B16-F10 cells remained close to Control at 0 h after treatment, while NeMBRT groups gave rise to spectral variations allowing them to be differentiated from the previous groups. At 24h post-RT, differences due to NeBB caused this group to separate further from the non-irradiated cells. However, NeMBRT-treated samples remained well distinct from the Control group, particularly for 8 Gy, with apparent differences between all irradiation configurations.

Furthermore, a difference in the clustering of RT modalities was observed for both cell lines one day after irradiations: NeMBRT clusters were always more proximate to Control than the NeBB group for the healthy cell line, whereas the three irradiation configurations remained differentiated from Control for the tumour cells, especially the BB and MB_peak_ groups.

#### HW region

Fig. 4 (bottom, left; 2 Gy and 8 Gy) and Fig. S1† (bottom, right; 4 Gy) show how BJ cells exhibit similar trends for the three studied doses at 24h post-RT. RT-treated cells are separating from Control due to changes in the four C–H stretching modes present in this spectral region. The biggest cluster delineation is encountered for the highest dose: RT-treated samples are differentiated from the Control group, particularly the NeBB cluster, and slight differences between MB_peak_ and MB_valley_ groups can be perceived. For the 2 Gy and 4 Gy, the main spectral bands accounting for the segregation of NeMBRT groups from NeBB are associated to the CH_2_ vibrational modes. Modifications of these bands also correlate with the differences between both NeMBRT peak and valley for 8 Gy, being the variations in the CH_3_ modes the ones that explain the separation of NeBB from the rest of samples. In particular, an increase of the asCH_2_/asCH_3_ spectral band ratio was observed due to NeBB for this dose (Fig. 5, top), suggesting that the length of acyl chains present in the cellular lipids increased after NeBB;^46^ other authors did also observe this behaviour and correlated it with cell death.^19^ For the other doses, irradiated configurations resulted in an slight intensity decrease of such spectral band ratio compared to Control, which might correlate with oxidative stress processes due to ROS attacks, affecting the saturation levels of acyl chains and phospholipid membranes.^32,45^

In this spectral region, the effects of the RT modalities on the IR spectra of BJ cells fixated at the two time-points were similar. For 2 Gy, the most different group from Control at 0h post-RT was NeBB, with NeMBRT groups being closer to untreated cells, but one day after RT they also became distinguishable from the Control group. For 4 Gy, RT-treated cells were always well distinct from non-irradiated cells, but 24h after treatment slight differences between NeMBRT groups and NeBB emerged. Lastly, for 8 Gy, NeMBRT clusters clearly separated from Control and NeBB groups just after treatment; at 24h post-RT, NeBB was also distinct from Control and NeMBRT groups, emerging differences between MB_peak_ and MB_valley_ groups as well.

Differences in the IR spectral features of B16-F10 cells subjected to the different RT modalities are also noticeable in Fig. 4 (bottom, right; 2 Gy and 8 Gy) and Fig. S1† (bottom, right; 4 Gy). For the lowest dose, there is an overlap between NeBB and NeMBRT groups, which separate from the Control cluster. For the intermediate and high doses, the four data clusters are well-separated, with the MB_peak_ being the most dissimilar group from Control. The most important contributions separating non-irradiated from irradiated groups come from the asCH_3_, asCH_2_ and sCH_2_ stretching modes. Differences in the asCH_2_ and sCH_2_ IR bands also allow to differentiate between BB, MB_peak_ and MB_valley_ groups. Higher absorbances of the methylene bands, along with a concomitant decrease of the CH_3_ stretching modes, resulted in an increase of the asCH_2_/asCH_3_ spectral band ratio (Fig. 5, bottom) for the irradiated groups, especially for the BB and MB_peak_. This behaviour suggests the activation of oxidative stress processes leading to the observed structural alterations. This would also explain the differences between BB and MB_peak_ with respect to MB_valley_, since in the high-LET regions of heavy ion beams a greater recombination of certain ROS occurs (in particular, of hydrogen peroxideH_2_O_2_). Therefore, the concentration of H_2_O_2_ in the valleys would decrease with respect to that in the peaks, resulting in less H_2_O_2_-induced damage (often considered a good candidate to explain MBRT efficacy).^17,48^ This behaviour is also consistent with our previous results observed for pMBRT in a rat glioma cell line.^25^

In this spectral region, NeMBRT appeared to induce greater effects on the C–H bands of B16-F10 cells than NeBB early after treatment, reflected by the separation of the groups at that time-point. But one day after RT, NeBB-treated tumour cells also underwent modifications that resulted in their separation from the Control group; despite this, NeMBRT still showed the largest IR differences compared to non-irradiated cells, particularly the MB_peak_ group.

## 4 Conclusions

In this study, SR-FTIRM allowed to assess the biochemical response of healthy (BJ) and tumour (B16-F10) cell lines to NeMBRT, a promising novel RT approach that combines the superior radiobiological properties of neon ions with the normal-tissue protection effects of MBRT. The use of synchrotron infrared light allowed to provide key information about subtle modifications in IR bands associated to lipids, proteins, nucleic acids and carbohydrates at the single cell level with a high signal-to-noise ratio. Despite the limited availability of synchrotron sources, this work is a relevant proof-of-concept study in view of the future availability of laser-based sources covering the mid-IR range.

Multivariate data analysis methods enabled to uncover the differential effects of NeBB and NeMBRT on both cell lines at 0h and 24h post-treatment. In general, the early impact of NeMBRT on the IR signatures of samples resulted in greater dissimilarities from the spectral pattern of the non-irradiated group than those due to NeBB, for both cell lines. NeMBRT-induced alterations in the 1800–1000 cm^−1^ spectral region might result from protein oxidation, nucleic acid rearrangements and/or oxidative stress early after irradiations; modifications of lipid-related spectral bands were also suggestive of lipid chain conformations or ROS attacks, especially in the tumour cell line. Nonetheless, the role of the repair mechanisms that NeMBRT has already been shown to activate was evident 24h after treatment: the IR signatures of the healthy samples subjected to NeMBRT were closer to those of the Control group. On the other hand, NeBB-treated healthy cells presented the most differing IR characteristics from the non-irradiated sample at this time-point, showing that NeBB-induced damage still persisted one day following RT; spectral alterations due to NeBB were consistent with enhanced oxidative stress. In contrast, the impact of both treatment modalities on the tumour cell line was more similar at 24h post-RT: the spectral features associated to lipids, proteins, nucleic acids and carbohydrates were highly affected by the BB and MB_peak_ groups at this time-point, with the latter configuration generally being the most dissimilar from Control. Modifications of the C=O groups of the proteins and the C–H bonds present in the phospholipids, mainly by the BB and MB_peak_ configurations, may be related to protein oxidation mechanisms, oxidative damage or cell death processes. However, further biological studies would be necessary to fully disentangle the radiobiological rationale underlying NeMBRT.

## Author contributions

RGV: formal analysis, investigation, visualization, writing (original draft); OS: investigation; AB: investigation; JB: investigation; RH: investigation; TI: investigation; NM: investigation; TS: investigation; YP: conceptualization, methodology, investigation, funding acquisition; IY: conceptualization, methodology, investigation, funding acquisition, writing (review and edit); IMR: project administration, conceptualization, methodology, investigation, funding acquisition, validation, data curation, writing (original draft). All authors reviewed the manuscript.

## Data availability

Research data will be stored and made available in the CORA research data repository (https://dataverse.csuc.cat).

## Conflicts of interest

The authors have no conflicts of interest to disclose.

## Supplementary Material

AN-150-D4AN01038H-s001
