# Correction: Synchrotron-based infrared microspectroscopy unveils the biomolecular response of healthy and tumour cell lines to neon minibeam radiation therapy

**DOI:** 10.1039/d5an90081f

**Published:** 2025-11-27

**Authors:** R. González-Vegas, O. Seksek, A. Bertho, J. Bergs, R. Hirayama, T. Inaniwa, N. Matsufuji, T. Shimokawa, Y. Prezado, I. Yousef, I. Martínez-Rovira

**Affiliations:** a Physics Department, Universitat Autònoma de Barcelona (UAB) 08193 Cerdanyola del Vallès Barcelona Spain Immaculada.Martinez@uab.cat; b IJCLab, French National Centre for Scientific Research 91450 Orsay France; c Institut Curie, Université PSL, CNRS UMR3347, Inserm U1021, Signalisation Radiobiologie et Cancer 91400 Orsay France; d Université Paris-Saclay, CNRS UMR3347, Inserm U1021, Signalisation Radiobiologie et Cancer 91400 Orsay France; e Radiology Department, Charité-Universitätsmedizin Berlin 10117 Berlin Germany; f Department of Charged Particle Therapy Research, Institute for Quantum Medical Science, National Institutes for Quantum Science and Technology (QST) 4-9-1 Anagawa Inage-ku Chiba-shi 263-8555 Japan; g Department of Accelerator and Medical Physics, QST 4-9-1 Anagawa Inage-ku Chiba-shi 263-8555 Japan; h New Approaches in Radiotherapy Lab, Center for Research in Molecular Medicine and Chronic Diseases (CIMUS), Instituto de Investigación Sanitaria de Santiago de Compostela (IDIS), University of Santiago de Compostela Santiago de Compostela A Coruña 15706 Spain; i Oportunius Program, Galician Agency of Innovation (GAIN), Xunta de Galicia Santiago de Compostela A Coruña Spain; j MIRAS Beamline, ALBA Synchrotron 08209 Cerdanyola del Vallès Barcelona Spain

## Abstract

Correction for ‘Synchrotron-based infrared microspectroscopy unveils the biomolecular response of healthy and tumour cell lines to neon minibeam radiation therapy’ by R. González-Vegas *et al.*, *Analyst*, 2025, **150**, 342–352, https://doi.org/10.1039/D4AN01038H.

In Fig. 2, the labels for asCH_2_ and asCH_3_ are interchanged, as well as the labels for sCH_2_ and sCH_3_ (HW region, 3000–2800 cm^−1^). The corrected version of the figure is presented here.

**Fig. 1:**
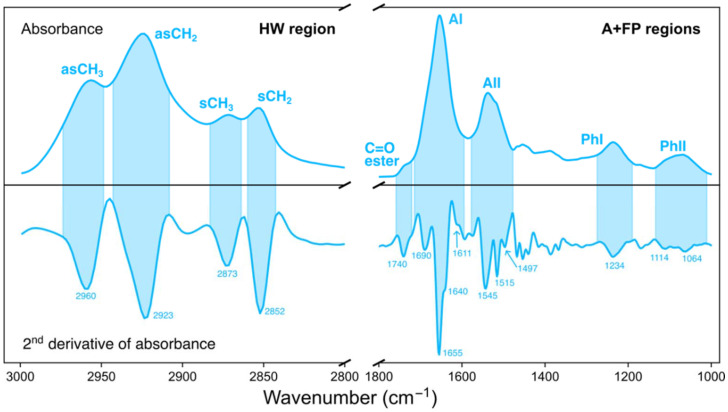
IR absorbance spectrum (top) of a cell and its second derivative (bottom) in the HW (left) and A + FP (right) spectral regions. Coloured areas indicate the spectral range of the indicated IR bands for both spectra. The positions (in cm^−1^) of the minima of the most relevant IR bands are indicated. The absorbance spectrum was baseline corrected and vector normalised; the second derivative spectrum was vector normalised.

The Royal Society of Chemistry apologises for these errors and any consequent inconvenience to authors and readers.

