# Supplementary material for: Synchrotron-based infrared microspectroscopy unveils the biomolecular response of healthy and tumour cell lines to neon minibeam radiation therapy
Source: Analyst. 2024 Dec 13;150(2):342–52. doi: 10.1039/d4an01038h (PMC11638702; doi:10.1039/d4an01038h)
Supplement: AN-150-D4AN01038H-s001 [file AN-150-D4AN01038H-s001.pdf]

Supplementary Information (SI) for Analyst.
This journal is © The Royal Society of Chemistry 2024

Supplementary Information

Synchrotron-based infrared microspectroscopy unveils the biomolecular response of healthy and tumour cell lines to neon minibeam radiation therapy

Roberto González-Vegas[1], Olivier Seksek[2], Annaïg Bertho[3,4], Judith Bergs[5], Ryoichi Hirayama[6], Taku Inaniwa[6,7], Naruhiro Matsufuji[6,7], Takashi Shimokawa[6,7], Yolanda Prezado[3,4,8,9], Ibraheem Yousef[10], and Immaculada Martínez-Rovira[1,*]

[1]*Physics Department, Universitat Autònoma de Barcelona (UAB), 08193 Cerdanyola del Vallès, Barcelona, Spain*

[2]*IJCLab, French National Centre for Scientific Research, 91450 Orsay, France*

[3]*Institut Curie, Université PSL, CNRS UMR3347, Inserm U1021, Signalisation Radiobiologie et Cancer, 91400 Orsay, France*

[4]*Université Paris-Saclay, CNRS UMR3347, Inserm U1021, Signalisation Radiobiologie et Cancer, 91400 Orsay, France*

[5]*Radiology Department, Charité-Universitätsmedizin Berlin, 10117 Berlin, Germany*

[6]*Department of Charged Particle Therapy Research, Institute for Quantum Medical Science, National Institutes for Quantum Science and Technology (QST), 4-9-1 Anagawa, Inage-ku, Chiba-shi 263-8555, Japan*

[7]*Department of Accelerator and Medical Physics, QST, 4-9-1 Anagawa, Inage-ku, Chiba-shi 263-8555, Japan*

[8]*New Approaches in Radiotherapy Lab, Center for Research in Molecular Medicine and Chronic Diseases (CIMUS), Instituto de Investigación Sanitaria de Santiago de Compostela (IDIS), University of Santiago de Compostela, Santiago de Compostela, A Coruña 15706, Spain*

[9]*Oportunius Program, Galician Agency of Innovation (GAIN), Xunta de Galicia, Santiago de Compostela, A Coruña, Spain*

[10]*MIRAS Beamline, ALBA Synchrotron, 08209 Cerdanyola del Vallès, Barcelona, Spain*

[*]Corresponding author. E-mail address: Immaculada.Martinez@uab.cat.

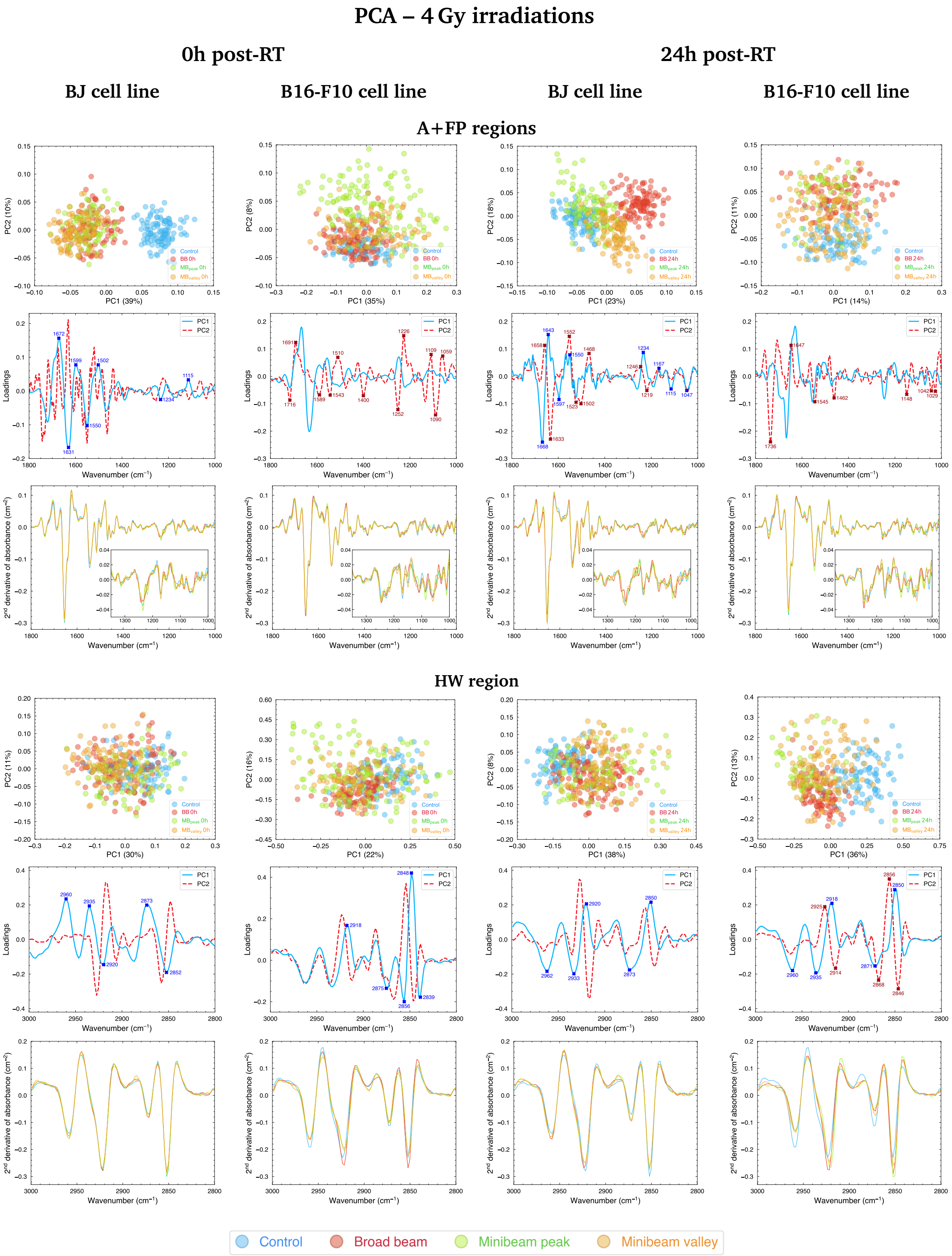

Figure S1. PCA in the A+FP (1800–1000 cm^{-1}, top) and HW (3000–2800 cm^{-1}, bottom) spectral regions for cell lines irradiated with 4 Gy and fixated at 0h (left) and 24h (right) post-treatment. For each spectral region and fixation time-point, the PCA scores (upper row), loadings (middle row) and average second-derivative absorbance spectra (lower row) are included for the BJ (left column) and B16-F10 (right column) cell lines. Each point of the PCA scores is a cell spectrum, and colours correspond to the irradiation configurations: blue for Control (non-irradiated), red for BB, green for MB_{peak} and orange for MB_{valley}. Explained variances by the PCs are included in parentheses. In the loadings, the contribution of each spectral band to data separation along PC1 is indicated by solid blue lines, while the bands contributing to the separation along PC2 are indicated by dashed red lines. The most relevant IR peaks contributing to the cluster delineation along PC1 or PC2 are indicated with blue or red labels and crosses, respectively. Indicated doses refer to the mean dose for both NeBB and NeMBRT configurations.

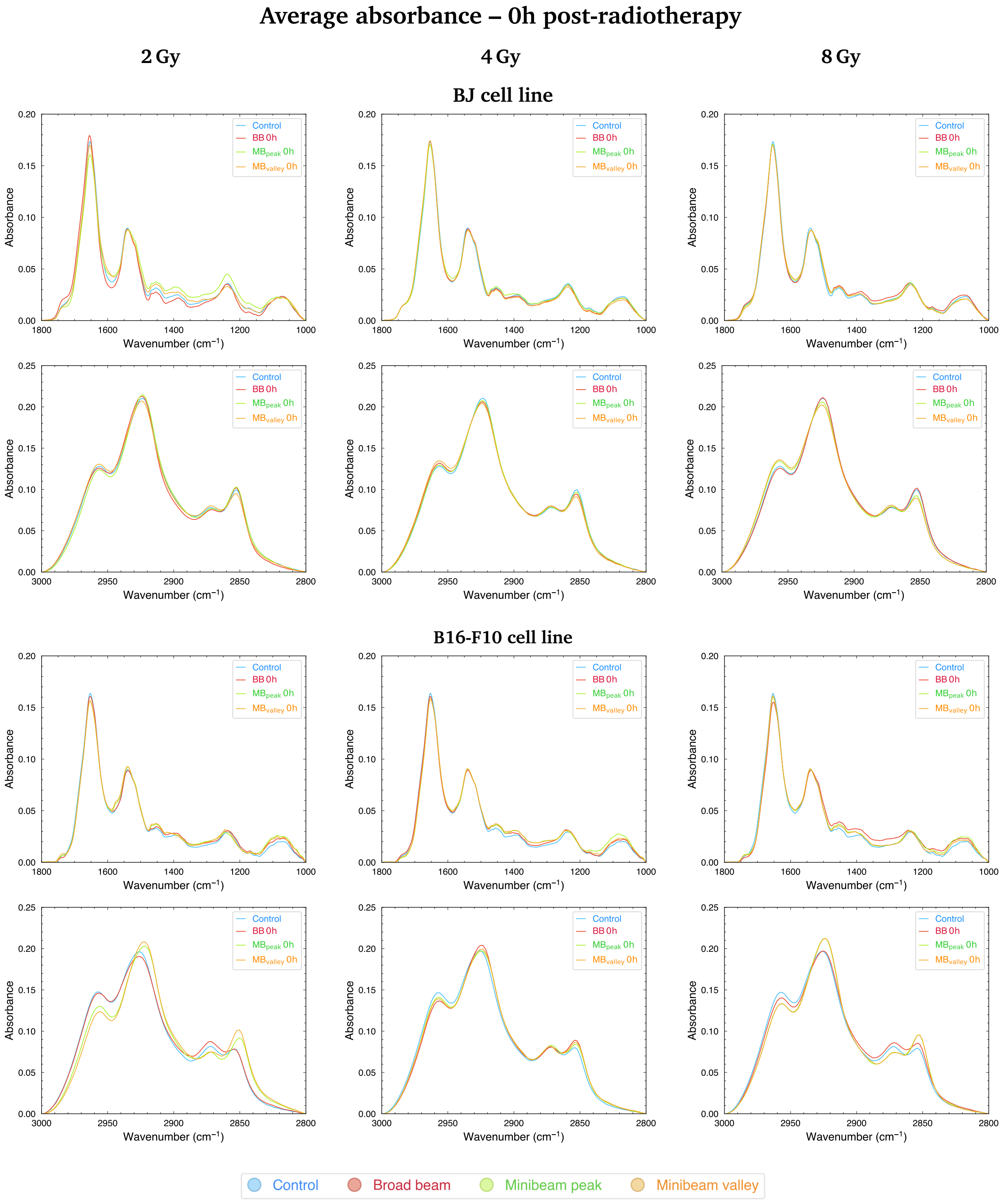

Figure S2. Average absorbance spectra of BJ (top) and B16-F10 (bottom) cell lines fixated at 0h post-RT. For each cell line, average absorbance spectra in the A+FP (1800–1000 cm^{-1}, upper row) and HW (3000–2800 cm^{-1}, lower row) spectral regions are included. Colours correspond to the irradiation configurations: blue for Control (non-irradiated), red for BB, green for MB_{peak} and orange for MB_{valley}. Indicated doses refer to the mean dose for both NeBB and NeMBRT configurations.

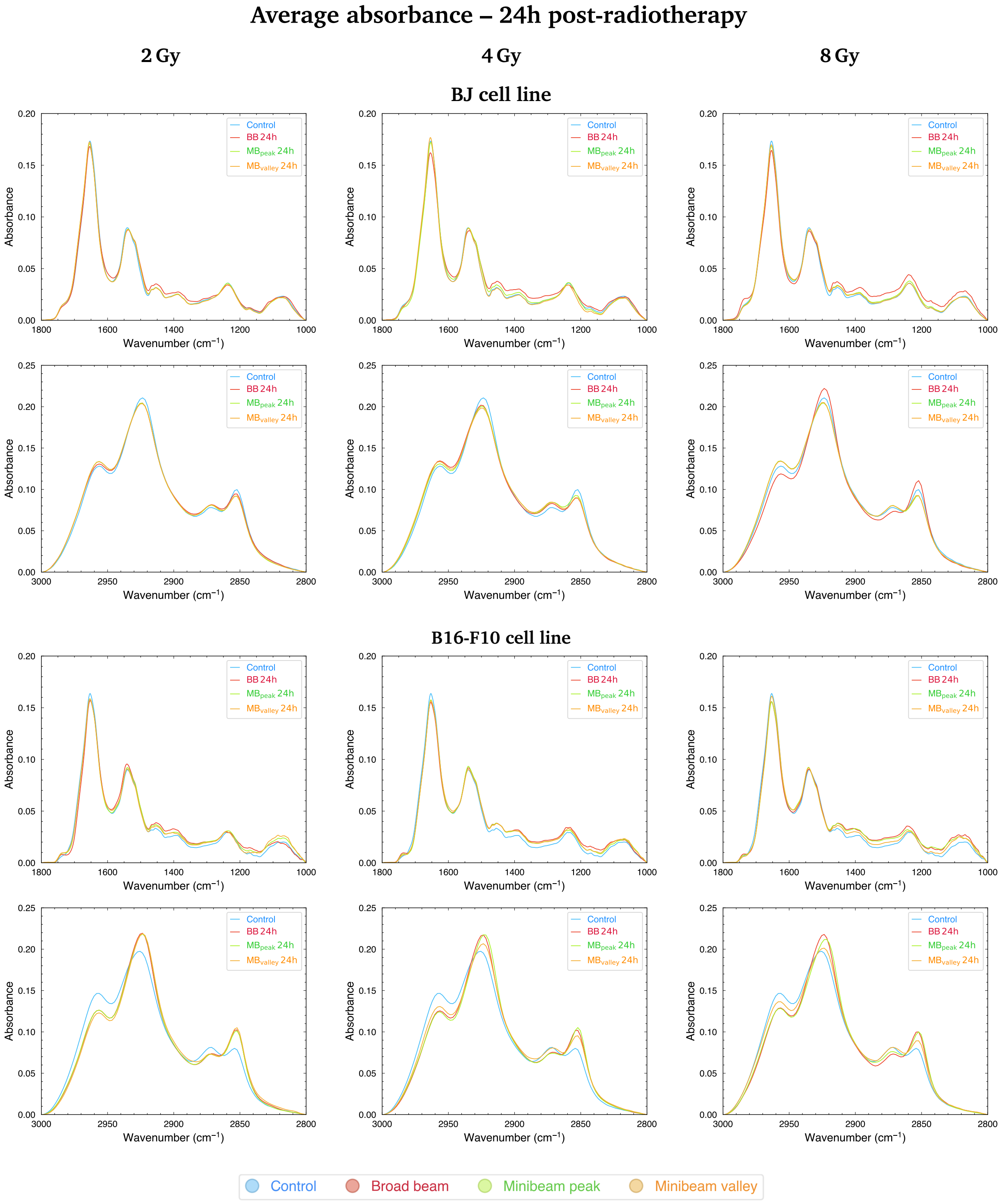

Figure S3. Average absorbance spectra of BJ (top) and B16-F10 (bottom) cell lines fixated at 24h post-RT. For each cell line, average absorbance spectra in the A+FP (1800–1000 cm^{-1}, upper row) and HW (3000–2800 cm^{-1}, lower row) spectral regions are included. Colours correspond to the irradiation configurations: blue for Control (non-irradiated), red for BB, green for MB_{peak} and orange for MB_{valley}. Indicated doses refer to the mean dose for both NeBB and NeMBRT configurations.
